# On Certain Microscopic Elements in Pulpless and Gum-Denuded Teeth, in Their Relations to the Filling of Roots, and the Re-Attachment of the Gum-Tissue

**Published:** 1884-03

**Authors:** J. Edw. Line

**Affiliations:** Rochester, N. Y.


### THE DENTAL REGISTER.

VOL. XXXVIII.] MARCH, 1884. [No. 3.

### COMMUNICATIONS.

### On Certain Microscopic Elements in Pulpless and Gum-Denuned Teeth, in Their Relations to the Filling of Roots, and the Re-Attachment of the Gum-Tissue.

BY J. EDW. LINE, D.D.S., M.D.S., ROCHESTER, N. Y.

Since the discovery by Spooner that arsenious acid properly applied to the tooth-pulp would devitalize that organ, the filling of root-canals has occupied a somewhat conspicuous place in dental theory and practice. The discovery in question is said by some to mark an epoch in the history of our calling, being regarded as the dividing line between old or ancient dentistry and modern or new. But if this discovery had ended simply in the destruction of the tooth-pulp, little would have been gained beyond the cure of tooth-ache without extraction and consequent loss of the tooth; and very much would have been lost in the rapid breaking down of the now poorly nourished tooth, the destruction of the osseous support of neighboring teeth, the drainage of the system by abscesses, and the poisoning of the oral secretions—and indirectly of the whole body—by the products of these outlets of badness. Happily it did not end here. The devitalization of the tooth-pulp was followed by its removal, whole or in part, and the more or less complete filling of the root canal, after or without medication, until the perfect filling of this cavity came to be regarded as a fine art.

The material of root-filling is not so important as formerly; the method of much more importance. Some were content to fill with cotton saturated with creosote or carbolic acid; some with cotton whose meshes had been filled with oxychloride of zinc; some with this only, or a similar plastic; some with gutta-percha; some with metals, as lead, tin, or gold, the latter ribbon-like or in roll-form, or wire, or some combination of the three. It was agreed to by good and bad operators alike that roots should be filled; that they should be filled to the apex; not beyond, for that would irritate the tissues of the alveolus and give rise to tenderness to touch, or possibly to inflammation and its various endings; not short of the apex, for thus would be left a pocket to hold shreds of pulp that might soon end in death, or to retain the juices of the neighboring tissues till time, and other circumstances favored their decomposition, when would result the many too well known ill effects.

The expert at root-filling is confronted with a case, say, of soreness of an incisor. The slight off-color, and other circumstances, indicate death of the pulp. Casual examination reveals, a gold filling on one side. Our expert now begins his quiz: "I see this tooth is filled; when was it done?" "Ten years ago." "By whom?" "Dr. Blank." "An excellent piece of work. Did he destroy the pulp?" "Yes." "And fill the root?" "Yes." Our expert is now suspicious of a defective root filling. Dr. Blank must have filled short of the end of the root, or beyond that point, or if just to the end his materials must have been poorly selected and as poorly packed. He then removes the filling from the cavity of decay and drills out the root-filling, or because of the worthlessness of the tooth's associates, or its own without them, extracts it and finds a root-filling as solid and as perfectly made as possible with soft gold foil packed by hand or by automatic mallet—a root-filling that goes just to, not beyond nor short of, the end. He pronounces it a perfect piece of work, and as he believes that roots perfectly filled never give trouble, he is puzzled. And well he may be, for within an hour of the operation in question he is called upon to extract, preparatory to the introduction of a plate, four pulpless incisors imbedded in healthy gums and process. On splitting these teeth for examination, as it is custom, he finds incisor A to contain the ideal root filling, satisfactory in every particular even to the odor of carbolic acid. Incisor B also contains a root-filling, but this falls short of the end, and yet there are no signs of pericemental trouble whatever. Incisor C is also filled, but in this the filling extends beyond the end of the root a full quarter of an inch. But there is nothing more out of the way in this than in cases A and B. Incisor D contains no filling at all, and as in the three already mentioned, indications of disturbance of whatever kind at or near apex of the root are wholly absent. If his reasoning is confined to these four teeth he concludes that a defective root-filling, or no filling at all, is just as good as the best. Or he may conclude that there is something beyond the antiseptically treated and perfectly mechanically filled root that has hitherto escaped his observation. And in this he is more than likely to be right; for while the making of the root thoroughly antiseptic and the filling of it in a mechanically perfect manner are to be desired, even if not necessary to success, there are other things that sooner or later undo the best that can be done while roots remain in their places in the jaw. What these things are will appear further on.

Whether the result of purely mechanical causes, as the tooth-brush or an ill-fitting partial plate; or the attendant or follower of causes pathological, as a low inflammatory, a suppurative or necrotic condition, traceable to deposits of tartar or constitutional peculiarities, the stripping of the teeth of their gum-tissue means death to the more superficial and exposed portions. It has been claimed time and again, publicly by the best and in a quiet way by some of the worst men in the profession, that the reproduction of the gum-tissue and its attachment to the tooth, or its attachment and the re-attachment of tissue already on the ground are matters of easy accomplishment, requiring merely time, patience, and skill. The best men above mentioned do this, or claim to do it, by surgical means chiefly; while others, the baser or basest extremists, rely on the application of medicaments known to domestic medicine and prescribed to innocents ever since coster-mongers ceased to jump upon their mothers. We are more than willing to admit the ability of any one of fair skill in other things, having a reasonably healthful and consequently hopeful case, to reproduce more or less gum-tissue, and by no other means than time, skill, and a well-formed lancet. On the other hand, we are but sparingly willing to admit that in the treatment of pyorrhœa alveolaris, or Riggs' disease, some are able not only to reproduce this tissue, but also to effect its union with diseased and gum-denuded teeth. We are thus "but sparingly willing" because we believe in many of these men, in their ability, their honesty, to report faithfully what they see, or think they see; and yet it is no more than fair that something more than mere statement, or say-so—something approaching demonstration should make its appearance, if only to bear these worthies out in what they have said in meeting and published in the journal. We do not say that the re-attachment of the gum-tissue to the tooth is impossible of accomplishment, that it cannot be done, that it has not been done; but we do say that there are certain things and conditions of things in the way that make the operation difficult and success doubtful. What these things are, and they do not differ in any essential from those hinted at in our remarks on root-filling, will be stated when we have considered for a moment the minute anatomy of the human tooth, which we now proceed to do.

According to the every-day notion of tooth-structure, a tooth is a mass of inorganic matter traversed in given directions by matter not inorganic, as in enamel; and in places and at times organized, as in dentine and cement. According to recent views, some of which are destined to rapidly and effectively supersede many now current, a tooth is an organized body having a lace or mesh-like structure, the meshes being filled in with inorganic matter, principally salts of lime. If from such a body a transverse section be cut just above the highest point of the enamel, and prepared for microscopical examination, there will be seen radiating from just within the pulp cavity numerous well-defined, nucleated cells, closely connected with each other by so-called lateral processes and sending one or more long, slender processes, or soft-fibers of Tomes, toward, into, and through the dentinal tubuli. If one of these processes or fibers be examined, lateral or sub-processes will be seen running towards and to neighboring processes, and by means of which all primary processes or fibers are bound together. If followed straight on this primary process, or soft fiber of Tomes, will be seen to unite directly with one or more processes of the cell of a cemental lacuna, or indirectly by processes as fine as those sent off from the sides. Following the process of the cemental cell we pass through the nucleus or nuclei, then through one of its peripheral processes, and finally through the mesh-like structure of other cells to the peridental membrane. This gives us a string of tissue connecting the pulp through dentine and cement with the membrane lining the alveolus; and this string of tissue will exhibit two or more nuclei or centers of assimilation, development, and (in early tooth history) reproduction—the three cardinal characteristics of organization, no matter where found. Such being the anatomy of this part of the tooth, of course roughly stated, we are prepared to go further and discover if possible some of the changes that may occur, and in occurring bring to naught all that has been done and suffered by operator and patient respectively.

If the pulp of a tooth die or be destroyed, the first thing is to remove the remains, cleanse the root to the apex, render it antiseptic so far as may be, and fill well from the end down. Suppose this done and the root does well for say ten years, as in our expert's case, and then, without apparent cause or provocation, becomes tender, aches, and after a few days develops an alveolar abscess. To what shall the failure be attributed? To mechanical interference of any kind? To accident? Neither of these. To the operator? No, for we know that he did his work well. What then? The answer is short: Death and decomposition of the contents of the dentinal tubuli, whole or in part, and the transmission of the irritation to and through the cemental cells to the peridental membrane, giving rise to inflammation and ending in abscess. It was at or near the pulp cavity that the trouble began, and it is here that a section from a tooth of the character described shows a difference in color, in the contents of the tubuli, in refractive power; and it is here that we find the line of demarkation, that line that sets off the living from the dead—what may be called the dead line. It existed at the time of the preparation of the root for filling. On one side was living tissue, on the other more or less dead tissue, antiseptically soaked. But at the time in question death took a fresh start and set the line deeper in the dentine, or even in the cement close to the peridental membrane, and with the result stated. Without knowing why, attempts have been to defer or prevent decomposition. The habit some dentists have of reaming roots from beginning to end is a good one, for by this means the dead contents of the inner ends of the tubuli and the diseased intertubular tis-ue are removed; but even then the dead line may be a little beyond the reamed portion—just far enough to save a little dead material for future mischief.

Again, in the case of a gum-denuded tooth, we find dead tissue near the surface of the root, and, in some cases, so far into the cementum as to involve the contents of the lacunæ. This being the case what is to bridge the chasm between the gums, old or recently formed, and the contents of the lacunæ or (in the neck) the tubuli? It is claimed that the root should be scraped, cut away, excised, and the gum lacerated, and then there will occur an exudation of plastic material which, undergoing organization, will re-establish nutritive and other relations between the tooth and its environment. Where this happens circumstances must be favorable indeed. That it does not, cannot, happen often is apparent from the fact that a broad dead line, a chasm must be crossed; and living tissue separated by such lowly organized structures as cement and dentine, and a thick film of mixed oral secretion, can rarely, if ever, come in contact—and union without contact of some kind and some thing is simply impossible.

In presentiug these thoughts it is not with the intention of excusing in any way any man for doing questionable work, but rather to put him on his guard against those who, in the face of the nature of things, claim too much; and also to furuish him with a few facts in regard to things always beyond his control and liable at any time to undo what has cost him long hours of hard and honest labor.

### DISCUSSION.

BODECKER, C. F. W.—I wish to say a few words with regard to the minute anatomy of the tooth. Dr. Line stated that the outer or peripheral surface of the pulp is covered by the odontoblast layer. This is indeed so; but he also said there was connection directly with the Tomes fiber—that the Tomes fiber springs directly from one end of the odontoblast. I have never been able to see this, and one who is accustomed to microscopical work who will take the trouble to make a section thin enough, and examine it with a power of at least 500 or 800, will be able to see very clearly, that the Tomes fiber does not arise from the end of the odontoblast, but between them. Whenever you come across a fiber which appears as though arising from the end of the odontoblast, if you will very carefully use the fine adjustment you will find it to come from a layer below. The Tomes fibers, as I have observed, originate directly from the lateral off-shoot to which they are connected, and to these again you may trace the ends of the non-medullated nerve fibers of the pulp. The doctor referred to the use of arsenious acid. I very much regret that I have been prevented by sickness this winter, as I would have presented a somewhat exhaustive paper to this Society on the action of arsenious acid. However, this has not been completed. Thus far I think the action of arsenious acid is not fully understood by any one, not even myself. I have experimented over two years, and sometimes it looked as though I had arrived at definite conclusions; but it seemed again and again that arsenious acid acted in this way, and sometimes in another. But the minute action of arsenious acid is an extremely difficult thing to find out. I am experimenting with three or four cases which are not yet completed, which promise some results, and if I have more time during the summer I will follow it up closely. Dr. Line gave us his views about the irritation from root-fillings. Gentlemen I am of the opinion that metal root-fillings such as amalgam, or gold, or even tin, are injurious to any root. In the first place they are too good conductors of thermal changes, and in the second place they do not perfectly seal up the dental canaliculi. The most perfect root-filling that I have employed is a solution of guttapercha in chloroform. Previous to this I had used oxychloride of zinc, and the last two years I have been in the habit of using a solution of iodoform in ether, and this in alveolar abscess has the most remarkable action I have ever seen. Dr. Line said that pyorrhoea alveolaris, or Riggs' disease, could not be cured. Certainly, in the cases where the pulp of the tooth is dead. I think it is a failure every time; but as long as the pulp is alive I think there is a very fair chance of success. About ten years ago, when Dr. Atkinson announced his method of treatment, a patient came to me with the four lower incisors very loose and a good deal of tarter on them, and asked: "What can you do?" I told him, nothing but clean and let alone; that they would probably drop out and that would be the end of it. He said: I don't want to lose my teeth. I want you to do something with them. I will give you a few months' time to think about it, but you must then do something with them." After eighteen months the gentleman came back with the teeth in a worse condition than before. He repeated his demand. One tooth was so loose that he was afraid when he used it that it might drop out. I cleaned them again and used aromatic sulphuric acid. In cleaning one of the centrals it almost fell over. I took an impression and made a support out of a couple of gold wires which would keep the tooth in position, and I continued the treatment every other day, or every third day, and in the course of about three months those teeth were as tight as any teeth in his mouth. That gentleman I saw again about six or eight months ago, and those teeth are just as firm to-day as any one of the teeth of the gentlemen in this room.

Elmendorf, M. E.—Did you use anything besides the aromatic sulphuric acid?

Bodecker, C. F. W.—At that time I did not, but now I have fortunately found several other substances which are excellent. There is a preparation of coal tar called tartarate of chinoline. It is an astringent as well as a powerful antiseptic, and it is the best I have ever tried.

Rich, J. B.—Do you mean to say that the teeth were absolutely loose?

Bodecker, C. F. W —They were absolutely loose.

Rich, J. B.—From the periosteum?

Bodecker, C. F. W.—I have now several other patients of the same character. If you wish to see them—

Rich, J. B.—You say the teeth were very loose, and I want to ask the question, Mr. President, of Dr. Bodecker, whether the periosteum was separated from those teeth at the time he commenced treatment?

Bodecker, C. F. W.—They were certainly separated at the neck. They were entirely loose.

Rich, J. B.—When those teeth became firm was it by the adhesion of the periosteum to the tooth again?

Bodecker, C. F. W.—That is what I believe

Rich, J. B.—Do you *know* whether it was or not? Did you make an examination?

Bodecker, C. F. W.—The gum has grown up tight to the tooth and consequently the periosteum.

Rich, J. B.—One would suppose that in a case so remarkable as this, a person who had been so successful in treating teeth under that peculiar condition, where they were very loose in the mouth and the periosteum entirely separated, would be very careful to ascertain whether the periosteum had become attached to the root again; because that is a question that with a great many men who are eminent in our midst, has been a strong point in dispute, whether that ever occurs or not. So far as I am concerned I am free to say I have never seen one single instance, where it was authenticated, where the periosteum became attached to the root and the tooth afterwards became firm.

Bodecker, C. F. W.—If Dr. Rich will come to the first clinic of the First District Dental Society, next month, I will have a case.

Rich, J. B.—I have seen cases, but never where this adhesion of the periosteum was identified—where the periosteum was thoroughly separated from the tooth, causing it to become loose, and where there was loss of the alveolus and the tooth afterwards became firm and solid.

Abbott, F.—That brings to mind one thing I wish to say, and that is, I have always fought the assertion that periosteum, when once destroyed by tartar, became restored. I had a case two or three months ago, however, which has shaken my position considerably. I examined it very carefully about a month after having thoroughly removed the tartar for the purpose of ascertaining whether it did occur (as a change had taken place), and to my astonishment I was thoroughly convinced that it had re-established itself, and re-attached itself to the tooth. So that where a month before I could put an instrument one-fourth of an inch between the gum and the tooth nearly all around the tooth, I could then find no detached point with the finest instrument I had at hand. The gum was in its original position. It was not denuded nor atrophied in the least. So I am convinced that restoration of the periosteum may be effected in some cases. It may be in the manner of treatment, It may be in the peculiar constitution of the patient. Our friend, Dr. Atkinson, has maintained for many years that the restoration of this membrane was a simple proceeding and often accomplished, which position I have as positively denied. Now I am ready to admit that it does occur once in a while. This is the only case, however, that I have ever seen. In reference to Dr. Line's paper, Dr. Bodecker has stated that any one who examined teeth carefully, who saw a "Tomes fiber," would readily observe it passing between the two odontoblasts as they presented themselves at the periphery of the pulp. I have seen it over and over again. The outer portions of the odontoblasts seem to be attached to and form a part of the fiber as it passes on into the dentine. In reference to the organic portion of the tooth after the death of the pulp; here we have an element that is ready to give us trouble at any moment. Dr. Line terms it very properly, a poison. It is a poison, if putrifying organic tissue is poison. This we find directly in connection with living organic tissue. I have called the attention of the profession in New York on several occasions to this fact, and have spoken of it as the possible cause of periostial irritation. When Dr. Jones or Smith or Brown has done a piece of work we know they have done it well. If the pulp canal has been treated and filled we are satisfied that it has been done properly, and yet we find in the cases treated by these gentlemen periostial irritation, starting up, frequently, without any explainable cause. It seems plain to me that under some circumstances there is a certain irritation of the living portion where it joins the dead and putrifying, by absorbing a portion of it, or something from it, by which irritation is conveyed through the cementum to the periosteum and thus periostitis is set up.

Rich, J. B.—I don't ask the question to be captious, but because it is a matter of great importance to the whole profession to know that as an absolute fact. From my standpoint I should not believe, except convinced by my own eyes, and my own senses. There is no proof except my own word of a very careful investigation of the treatment by which this periosteum was attached, and what it was attached to. Then, I should want to know, for the benefit of my profession, so we might combat this most dangerous of all diseases, that causes the destruction of so many splendid teeth, and I should want to place before the profession the means by which this miracle was performed, for I would regard it as a miracle even if I saw it. I have seen it asserted. I say why don't you show it to somebody before you begin the treatment, because, when you assert a thing that is doubtful, very few people believe, unless you allow several people to see. I would not believe the authority of any man unless I saw it, in cases of that kind, for it has been against the teachings of my whole professional life, and I have had a great many cases presented to me. I say I want to see this man, and why don't you give publicity to the treatment? Therefore, it was not in a captious spirit I asked this, but only because I want to know the facts; but I will go any distance where I can see both stages of the case. I want to see before the treatment commences, where I can examine for myself, and then I want to see it fastened when it is completed—when we can make an investigation and see if it is attached.

Atkinson, W. H.—I suppose I may say this is my fight. I claim to have no other object in stating my perception of the facts than the illumination of men who are in the condition I

once was. When we have been blessed ourselves, we want the whole world to be converted at once. While we are warm with the perception of the definiteness and usefulness of the truth that has been revealed to our consciousness, we are all awake, and come to the husking-bee. I know what I am saying, and have for years. I know what it is to be as earnest against the position as I am in the position I take now, and I know one is the position of light and the other is that of darkness. The fact is, that every revelation of truth to the human mind has to come through illumination of our obscurity to become light. Where is the difficulty? It is that men merely skim over the subject of embryonal conformation of tissue, and do not understand the ultimate physiology by which the tissues are repaired under the demand of typal proportion. I have said, time and time again, reproduction of tissues had to pass through the same stages, and steps, by the guidance of typal presence, as it did in original production. Stick a pin there, and hold your attention to the point. It is this: They have supposed periosteum once formed must be nourished intact, all its elements being continually fed, and if they were destroyed they could not be reproduced. If they studied the first line of the first letter of the alphabet that constitutes the A of histology, they would not make such a blunder. Every reproduction conforms to the plan of building up a new tissue upon the point in protoplasm that has been called a "vacuole," that is exactly like the original one in the development of the chick in the egg of the hen, or the human fœtus in the human egg in utero, that this metamorphosis takes place in the exudate of blood-plasm which is divided into the tissues of the body to complete the functioning machine. We know scar-tissue is built upon the type of the original connective tissue, at the point where the demand of this innocent thing we call type directs, so that the scar-tissue may be first class. This is exemplified in a case that occurred some fifteen years since, in which new alveolar processes were obtained where the old were necrosed and taken from the under jaw of a young lady, a student in Packer Institute, in Brooklyn. The dead processes were removed through slits in the swollen and inflamed gums, from the second molar on the right side to the second molar of the left side. The right inferior second bicuspid was extracted before the case was seen. Three of the teeth had dead pulps in them; but this did not preclude the formation of perfect processes and gums to the normal necks of the teeth, incisors, cuspids bicuspids, and one molar. The pulplesss teeth are very dark, but the attachment of the gums and the alveolar processes are indistinguishable from the originals. That individual is still living, and the case has been reported a number of times before this body and others. Yet some men, claiming to be intelligent students of histology and pathology, persistently deny these facts, and demand that we shall esteem their judgment at such a high value as to force us to seek out and give them opportunity to see every case we have. These are the men who stand with their back to the light and seeing their own shadow, at which they persistently look, say: "By God, it is dark!" This is a question of too much importance to the human family, and to us as agents of the blessed intent, to follow such darkness as brought us into this difficulty, and has made, by the force of circumstances, this noble body, before whom I stand with trembling earnestness, with tender affection and fearful as a fawn lest the tusks of the wild boar should rip out our bowels and make here an example of reproduction after the manner of the fakirs of Egypt. I want to speak to the points at issue, and thank Dr. Line for his paper. If men were able to digest that paper, we would not have such false diagnosis as we have. If men knew what they were about, and what their highest interest is, they would get down on their knees, if need be, and examine with earnestness and faithfulness the questions which are involved. And first, the doctor has made in the paper one grand mistake. He has assumed conditions that are impossible to the man who will go a little further than he has gone. He speaks of the action taking place in a perfectly sealed chamber, which is impossible. I question all the surgery in war and other places; I have seen magnificent examples this day in the museum, where bullets had been driven into the human body and encysted; but where they were driven and encysted all but a single fragment, which, by reason of cutting through the bone, a degree of inflammation had been set up, and reproduction by reason of the returning of the tissue to an embryonic condition. These patients lived a number of years, and died of disease. We can get a tooth encysted, whether the root is filled or not. That was told us years ago by our strong-headed Scotch friend, Watt, of Ohio. He didn't care whether the pulp-chamber was filled if the end was properly encysted, so as to cut off the diseased impact of energy. I can show cases that will verify the testimony to any person whose mind is in a teachable condition. I have a very cultured lady who had been in the care of a dentist who loved her as much as he could love a patient, and who said, when she insisted on not having the teeth out: "I will send you to a man who will do for you the best that can be done." The teeth were the first left inferior second molar, first molar and both bicuspids on the right side, and three molars on the left side below, and all these teeth were loose and their sockets were oozing pus all the time. I can give the name and letter of introduction, if need be, of the dentist, whom the lady reports as saying: "No man can save the teeth; but I will send you to a crazy man, who will take you in his lap and kiss you, and you will think he is almost divine, and you will come back to me in two or three years for artificial teeth." That is the kind of treatment I get from my best friends, and a man whom I tried to take from a shoemaker's bench to be a dentist. All the upper back teeth of one side were gone except one bicuspid. She was wearing black rubber with gold on the face of it to protect the gum. The teeth were loose and pus-discharging all around. It will be four years next November since that case came into my hands, with the mouth raw. I took care of it by simple treatment, beginning by tying the teeth together with floss silk, two or three lines of it, by reason of their being so frail.

Rich, J. B.—I rise to a point of order and that is, there is a great deal of the gentleman's remarks that is personal and does not enter into discussion before a scientific body.

Straw, President—The speaker will confine himself to the subject under discussion.

Atkinson, W. H.—When I aspire to put forth the truth which has cost me an earnest life-time to formulate, personality is a little matter, and an individual who has the truth within him is above or below misrepresentation, and you can use that to suit yourselves. But I am in such earnest to get this which has been revealed to me into the minds of men who can bless the world if they will follow the lead I am following. The covered mucous surface instead of discharging mucus was discharging pus and as raw as if denuded of the epithelium. I don't say the whole epithelium was gone, for then you could not get complete reproduction. Three weeks she was in my care and I put her in a Reese-metal-plate which is adapted to the conductivity of the tissues. And she has the third plate in three years by reason of the shrinkage of the parts. The treatment was cleaning off the foreign matter as far as I could find it, and then drying the tissues out as well as possible, and, with a little bit of wood dipped into aromatic sulphuric acid, full strength, drop in until every one of the little pockets were filled. Do it but once and I will leave you to your convictions; afterwards you will watch it day by day. If it heals by first intention don't over-meddle. There is as much mischief done by over-treatment as under-treatment, and I guess more. How? When nature has set the machinery to work and poured out pabulum inside this new pocket that is to be formed into the periosteum and other issues of the locality we should simply keep it clean. There is not a bit of pus in that lady's mouth except at the festoon of the gum at the mesial side of the right central incisor. The upper ones have a good calcified reproduction of the alveolar process, not a particle of discharge and no unhealthy appearance. I touched with salicylic acid (saturated solution in alcohol). That coagulates the albumen on the outside so as to make the equivalent of the *decidua reflexa* in gestation and re-production. That and other cases can be seen. The only secret is to keep foreign matter away and form the pocket so that the lips and tongue and food cannot come in contact with the new growth. When it is there the work is done. It does require to see whether you have removed all the debris and mischief, because here and there there may be a little spot that has been missed. If you are in earnest you will watch with an interest above the accumulation of dollars and cents.—*Transactions of the Dental Society of the State of New York.*

